# High level of clinical inertia in insulin initiation in type 2 diabetes across Central and South-Eastern Europe: insights from SITIP study

**DOI:** 10.1007/s00592-019-01346-1

**Published:** 2019-04-16

**Authors:** Matthew D. Campbell, Drazen Babic, Uros Bolcina, Lea Smirčić-Duvnjak, Tsvetalina Tankova, Asimina Mitrakou, Peter Kempler, Andrej Janez

**Affiliations:** 1 School of Food Science and Nutrition, University of Leeds, Leeds, UK; 2 Multidisciplinary Cardiovascular Disease Research Group, University of Leeds, Leeds, UK; 3 Diabetes Education and Research Institute AGADA, Ljubljana, Slovenia; 4 University Clinic for Diabetes, Endocrinology and Metabolic Diseases Vuk Vrhovac, Merkur University Hospital, Zagreb, Croatia; 5 Clinical Centre of Endocrinology, Medical University, Sofia, Bulgaria; 6 1st Department of Medicine, Semmelweis University, Budapest, Hungary; 7 Department of Endocrinology, Diabetes and Metabolic Diseases, University Medical Centre, Ljubljana, Slovenia

**Keywords:** Insulin therapy initiation, Type 2 diabetes mellitus, Clinical inertia

## Abstract

**Aims:**

Little is known regarding initiation of insulin therapy in type 2 diabetes (T2D) in Central and South-Eastern European countries. Therefore, we conducted a survey to characterise the prescribing practices of specialist diabetes healthcare professionals in this region and assessed factors that influence clinical decision-making regarding insulin initiation in T2D.

**Methods:**

A cross-sectional survey sampled 211 specialist diabetes healthcare prescribers from five Central and South-Eastern European countries (Bulgaria, Croatia, Greece, Hungary, and Slovenia). A structured questionnaire was developed which surveyed current clinical practices and influencing factors, barriers to insulin initiation, and combination therapy prescribing preferences.

**Result:**

Only 9.4% (20 of out of 211 respondents) of healthcare professionals would initiate insulin therapy in T2D patients at the recommended HbA1c threshold of 7–7.9% [53–63 mmol/mol]. Large regional differences were evident in insulin initiation thresholds (≥ 9.0% [≥ 75 mmol/mol]: Bulgaria 80.8% vs. Slovenia 13.3%). Psychological distress was recorded as the major barrier to insulin initiation. Health insurance regulations were ranked more important than personal clinical experience and clinical guidelines in clinical decision-making. Information from peers was more influential than manufacturer information, clinical experience, and continuous medical education, respectively, for insulin initiation.

**Conclusions:**

Despite large regional variation, there is widespread delay of insulin initiation from specialist diabetes healthcare professionals in Central and South-Eastern Europe.

**Electronic supplementary material:**

The online version of this article (10.1007/s00592-019-01346-1) contains supplementary material, which is available to authorized users.

## Introduction

Type 2 diabetes (T2D) is one of the greatest global health emergencies of today with almost a half a billion people living with the disease [1]. To lessen the burden of T2D, there is an urgent need for responsive, responsible, and effective clinical care. Through large clinical trials, it is understood that intensive glucose control prevents microvascular complications [2] and improves cardiovascular outcomes [3] in adults with T2D. In Europe, as well as the USA [4], clinical care guidelines stipulate early adoption of insulin and escalation as part of intensifying T2D treatment to aggressively lower glycated haemoglobin (HbA1c) below a general target of 7% (53 mmol/mol). However, it is also understood that insulin initiation is often inappropriately delayed [5], resulting in an unnecessary increased risk of complications and needlessly reduced life expectancy and quality of life [6]. This has been termed ‘clinical inertia,’ and can be due to a number of factors including clinical concerns (i.e. risk of weight gain, hypoglycaemia, or patient distress), professional concerns (e.g. lack of clinical experience, skills, or confidence in insulin titration), or health system concerns (competing priorities, regulatory or financial constraints, or a lack of impartial continued medical education [CME]) [7–13]. Recently, a systematic review highlighted the global widespread extent of clinical inertia in the management of hyperglycaemia in T2D including studies from the USA (29 studies), Europe (20 studies), and Asia (3 studies) [5]. However, available European studies typical sample from northern European countries including the UK, France, Spain, and the Netherlands [5], with only one study assessing clinical inertia from Central and South-Eastern European regions (one study in Croatia) [14]. Countries in Central and South-Eastern Europe compare poorly to countries in northern Europe due to a number of factors including different healthcare systems, treatment availability, and clinical training. As such, we aimed to comprehensively characterise the prescribing practices of specialist diabetes healthcare professionals across Central and South-Eastern European countries and assess the factors that influence their clinical decision-making regarding insulin initiation in T2D.

## Methods

The Study of Insulin Therapy Initiation in type 2 diabetes in routine clinical Practice (SITIP) is an ongoing multicentre international cross-sectional observational epidemiological study of factors which influence insulin initiation in type 2 diabetes. A cross-sectional survey was performed between September 2017 to January 2018, on 233 actively prescribing specialist diabetes healthcare professionals residing across five Central and South-Eastern European countries (Bulgaria, Croatia, Greece, Hungary, and Slovenia). Study participants were recruited through invitation from participating National Diabetes Associations as well as through the Advances in Diabetes and Insulin Therapy (ADIT) conference database. Following appropriate institutional ethical review, Informed consent was obtained from all individual participants included in the study. The sample (*n* = 211) represents 21.4% of all 987 prescribing specialist diabetes healthcare professionals within the region (Bulgaria *n* = 52 [24.9%]; Croatia *n* = 19 [9.0%]; Greece *n* = 51 [24.3%]; Hungary *n* = 73 [34.9%]; Slovenia *n* = 15 [6.9%]). Sample size was calculated with regard to confidence intervals (CI) for frequencies: a 95% CI of 10–15% for questions answerable was considered narrow enough. This required a sample size of ~ 200 persons. A structured 17-item closed multiple choice questionnaire was designed to collate population demographic information alongside prescription preferences, as well as habits and attitudes towards insulin initiation in T2D using Likert items; the questionnaire was devised by the research team and is shown in supplement 1.

### Statistical analysis

All analyses were performed with SPSS statistical software, version 23 (IBM SPSS Software, IBM Analytics), with significance set at *p* < 0.05. Descriptive statistics were mainly used with continuous variables (i.e. data collected using scaled parameters) and presented as mean ± standard deviation (SD), unless otherwise specified, and categorical data (i.e. prevalence) presented as absolute number and percentage. The Chi square test was used for comparisons of categorical data (Yates continuity corrected for comparisons with one degree of freedom). Two-sided *p* values of less than 0.05 were considered significant. With regards to continuous data, regional response rates were weighted to an appropriate share.

## Results

Remarkably, only 9.4% of healthcare professionals would initiate insulin therapy at the recommended HbA1c threshold of 7–7.9% ([53–63 mmol/mol]; Table 1). Almost half of the healthcare professionals (47.4%) would initiate insulin therapy at an HbA1c threshold of ≥ 9.0% (≥ 75 mmol/mol), and 8.0% would not initiate insulin until a threshold of ≥ 10.0% (≥ 86 mmol/mol) was reached (Table 1).

**Table 1:** Composite and regional data regarding clinical practice and influences of clinical practice in insulin initiation and alternative therapies in T2D

	Composite	Bulgaria	Croatia	Greece	Hungary	Slovenia	Test statistic
HbA1c							
7.0–7.9% [53–63 mmol/mol]	9.4%	1.9%	10.5%	19.2%	6.7%	13.3%	–
8.0–8.9% [64–74 mmol/mol]	43.2%	17.3%	47.4%	46.1%	52.0%	73.3%	–
9.0–9.9% [75–85 mmol/mol]	39.4%	77.0%	36.8%	28.9%	26.6%	13.3%	–
≥ 10.0% [≥ 86 mmol/mol]	8.0%	3.8%	5.3%	5.8%	14.7%	0.0%	–
Clinical barriers to insulin initiation							
Hypoglycaemia	1.84 ± 1.05	2.11 ± 1.21	1.80 ± 1.06	1.56 ± 0.89	1.85 ± 1.06	1.79 ± 0.79	*p* = 0.148
Weight gain	2.37 ± 0.94	2.33 ± 1.05	2.13 ± 0.87	2.63 ± 0.91	2.18 ± 0.78	2.83 ± 1.19	*p* = 0.011
Psychological distress	3.06 ± 1.04	2.65 ± 1.10	3.26 ± 0.92	2.98 ± 1.13	3.38 ± 0.86	2.86 ± 1.08	*p* = 0.001
Quality of life	2.74 ± 1.05	2.91 ± 0.96	2.80 ± 1.08	2.83 ± 0.97	2.58 ± 1.13	2.52 ± 1.17	*p* = 0.344
Factors influencing clinical decision-making							
Clinical guidelines	1.52 ± 0.71	1.54 ± 0.69	1.70 ± 0.83	1.34 ± 0.65	1.58 ± 0.73	1.62 ± 0.63	*p* = 0.135
Health insurance constraints	2.35 ± 0.79	2.28 ± 0.83	2.43 ± 0.79	2.71 ± 0.51	2.16 ± 0.85	2.17 ± 0.82	*p* = 0.004
Clinical experience	2.12 ± 0.72	2.17 ± 0.74	1.87 ± 0.70	1.95 ± 0.63	2.26 ± 0.70	2.21 ± 0.92	*p* = 0.136
Factors influencing insulin initiation practices							
Clinical experience	2.07 ± 0.91	2.22 ± 0.87	2.04 ± 1.05	2.02 ± 0.79	2.08 ± 1.00	1.72 ± 0.86	*p* = 0.201
Information from peers	3.14 ± 1.00	3.20 ± 1.09	3.20 ± 0.95	3.12 ± 1.05	3.16 ± 0.92	2.79 ± 1.00	*p* = 0.000
Manufacturer information	2.77 ± 1.07	2.57 ± 1.05	3.09 ± 0.88	2.88 ± 1.07	2.66 ± 1.12	3.21 ± 0.96	*p* = 0.714
CME education	2.02 ± 1.07	2.02 ± 1.12	1.67 ± 0.83	1.98 ± 1.08	2.09 ± 1.07	2.28 ± 1.18	*p* = 0.119
Approach to insulin initiation							
Basal insulin	78.6%	67.3%	88.8%	96.0%	73.0%	75.0%	–
Biphasic insulin	9.0%	25.0%	11.2%	2.0%	0%	18.7%	–
Prandial insulin	0.5%	1.9%	0%	0%	0%	0%	–
Basal-bolus	11.9%	5.8%	0%	2.0%	27.0%	6.3%	–

Note: Continuous data are presented as mean ± SD, and categorical data are presented as %

As shown in Table 1, when assessing data by region, 77.0% of respondents from Bulgaria would only initiate insulin therapy when an HbA1c threshold of ≥ 9.0% (≥ 75 mmol/mol) was reached, of which 96.2% would not initiate insulin until ≥ 10.0% (≥ 86 mmol/mol). In Hungary, 26.6% of respondents would only initiate insulin therapy at an HbA1c a threshold of ≥ 9.0% (≥ 75 mmol/mol), of which 85.3% would not initiate insulin until a threshold of ≥ 10.0% (≥ 86 mmol/mol) was reached. Conversely, in Slovenia, 13.3% of respondents would initiate insulin therapy at an HbA1c threshold of ≤ 9.0 (≤ 75 mmol/mol) and 13.3% would initiate insulin therapy at an HbA1c threshold of 7–7.9% (53–63 mmol/mol). Predominantly, basal insulin was primarily selected when initiating insulin therapy (78.6% of respondents), followed by basal-bolus (11.9%), biphasic (9.0%), and prandial (0.5%) insulin.

Psychological distress was recorded as the most prevalent barrier to insulin initiation across the region and risk of hypoglycaemia the least prevalent (Table 1); weight gain was considered secondary to quality of life as a barrier to insulin initiation. Factors influencing clinical decision-making were consistent across the region; health insurance regulations and constraints were listed as the most important determining factor. Conversely, clinical guidelines were the considered the least important factor influencing clinical decision-making (Table 1). The most important factor specifically influencing insulin initiation was information from peers, followed by manufacturer information, with CME ranked lowest.

## Discussion

This is the first study to comprehensively examine the prescribing practices and factors of influence of specialist diabetes healthcare professionals regarding insulin initiation in T2D across Central and South-Eastern European countries. In this large (*n* = 211) cohort, we show a high prevalence of clinical inertia, with only ~ 10% of prescribers initiating insulin therapy at the recommended HbA1c threshold of 7–7.9% (53–63 mmol/mol). Moreover, almost half the healthcare professionals sampled were orientated towards initiating insulin therapy in patients if an HbA1c threshold of ≥ 9.0% (≥ 75 mmol/mol) was met, with a further ~ 10% only initiating insulin when a threshold of ≥ 10.0% (≥ 86 mmol/mol) was reached. Further, we highlight substantial regional differences in the clinical approach to insulin initiation in T2D. For example, almost 80% of respondents from Bulgaria would only initiate insulin therapy in T2D at an HbA1c a threshold of ≥ 9.0% (≥ 75 mmol/mol), of which ~ 95% would not initiate insulin until a threshold of ≥ 10.0% (≥ 86 mmol/mol) was reached, compared to < 15% of prescribers in Slovenia. These results indicate that only the most poorly controlled patients, as opposed to all patients with evidence of inadequate glycaemic control, are likely to receive timely insulin initiation and that this is in part dependent upon country of residence. Our findings compliment and extend other large multinational studies reporting clinical inertia with regards to insulin initiation in T2D in Europe and elsewhere [15, 16].

Preference of basal insulin as the initial choice of insulin therapy is supported by the observation that basal insulin is often judged easier to use and with less risk of hypoglycaemia and weight gain. Further, although a premixed and a basal-bolus regimen have been found to yield greater reductions in HbA1c, compared to basal-only therapy, both are associated with weight gain, increased hypoglycaemia, and inconvenience [17, 18].

Previously, physician-, patient-, and healthcare system-related factors have been identified as contributing factors to clinical inertia in insulin initiation [19]. In our study, we identified consistent patterns in factors influencing clinical decision-making across the region. Our study participants ranked health insurance regulations and constraints as the most important factor influencing *prescription practices*, with clinical guidelines ranked as the least important factor. Further, patient distress was highlighted as the most important factor influencing *clinical decisions*, whereas risk of hypoglycaemia and potential weight gain as the least important factors. In the countries we sampled from, there is restricted regional availability of blood glucose self-monitoring (BGSM) reimbursement [20], and increasing BGSM increases the patient burden of managing T2D [21].

Our data indicates that healthcare professionals within the region turn primarily to peers and manufacturer information to guide and inform insulin initiation practices and that such practices are informed least from CME. Whereas some research has suggested that healthcare professionals perceive industry influence to be low [22], the reality is that the pharmaceutical industry is often a key source of information regarding new products and treatment options [23]. Indeed, several studies have shown that physicians are susceptible to the pharmaceutical industry and interactions with pharmaceutical sale representatives and that this influences prescribing practices [24–26]. In our study, we cannot rule out that the availability of increasing numbers of non-insulin antidiabetic agents and a lack of accessible independent CME could foster a reluctance to use insulin among our healthcare professionals. The concept of evidence-based medicine, defined as the integration of best research evidence with clinical expertise and patient values, is considered an integral part of medical training and should be effectively integrated into independent, non-industry sponsored CME courses [27]. Alarmingly however, our findings suggest that the majority of healthcare professionals within Central and South-Eastern European regions may not implement clinical guidelines and may not use evidence independent of industry to inform T2D treatment. As such, there is an urgent need to provide accessible CME courses independent of industry influence to prescribing specialist diabetes healthcare professionals within this region.

In conclusion, we provide new evidence which highlight a high level of clinical inertia regarding insulin initiation in T2D by prescribing diabetes specialist healthcare professionals in Central and South-Eastern Europe. We provide valuable insight into the factors influencing prescribing practices and clinical decision-making which highlight the urgent need to provide CME courses independent of industry in this region.

## Electronic supplementary material

Below is the link to the electronic supplementary material.

Supplementary material 1 (DOCX 14 kb)
