# Supplementary material for: High level of clinical inertia in insulin initiation in type 2 diabetes across Central and South-Eastern Europe: insights from SITIP study
Source: Acta Diabetol. 2019 Apr 16;56(9):1045–9. doi: 10.1007/s00592-019-01346-1 (PMC6675753; doi:10.1007/s00592-019-01346-1)
Supplement: Supplementary file 1 — Supplementary material 1 (DOCX 14 kb) [file 592_2019_1346_MOESM1_ESM.docx]

**SUPPLEMENT 1**

**SITIP STUDY QUESTIONNAIRE**

**Please complete all questions in full.**

**Personal and demographic information**

1. **What is your email?**
2. **What is your first name?**
3. **What is your last name?**
4. **What is your gender?**
   1. **Female**
   2. **Male**
5. **What is your age?**
   1. **18 to 24 years**
   2. **25 to 24 years**
   3. **35 to 44 years**
   4. **45 to 54 years**
   5. **55 to 64 years**
   6. **65 to 74 years**
   7. **75 years or older**
6. **In what country do you live?**
7. **Do you consent to be contacted to discuss your answers to this questionnaire?**

**Prescribing practices**

1. **Do you introduce insulin therapy to T2DM patients in your clinical practice?**
   1. **Yes**
   2. **No**
2. **On average, at which HbA1c level would you typically initiate insulin therapy in patients with T2DM in your clinical practice?**
   1. **7.0 - 7.9% [53 - 63 mmol/mol]**
   2. **8.0 - 8.9% [64 – 74 mmol/mol]**
   3. **9.0 - 9.9% [75 – 85 mmol/mol]**
   4. **≥10.0% [86 mmol/mol]**
3. **Which are your key concerns when initiating insulin insulin therapy in patietns with T1SM? Please rank options in order of preference; use 1 for your most preferred option and 4 for your least preferred option.**
   1. **Hypoglycaemia**
   2. **Weight gain**
   3. **Psychological distress**
   4. **Quality of life**
4. **Do you implement a comprehensive diabetes education programme for your patients with T2DM *before* initiating insulin therapy?**
   1. **Yes**
   2. **No**
   3. **Sometimes**
5. **Do you implement blood glucose self-monitoring (BGSM) in patients with T1DM *before* initiating insulin therapy?**
   1. **Yes**
   2. **No**
   3. **Sometimes**
6. **Do you find BGSM-derived data helpful when choosing insulin regimens for patients with T2DM?**
   1. **Yes**
   2. **No**
7. **Which is your most common approach to initiating insulin therapy in patients with T2DM?**
   1. **Basal insulin**
   2. **Biphasic insulin**
   3. **Prandial insulin**
   4. **Basal-bolus**
8. **Do you take patient preferences into account when choosing insulin regimens for patients with T2DM?**
   1. **Yes**
   2. **No**
   3. **Sometimes**
9. **When choosing insulin regimens for patients with T2DM, what factors influence your clinical decision making? Please rank options in order of preference; use 1 for your most preferred option and 3 for your least preferred option.**
   1. **Clinical guidelines**
   2. **Health insurance constraints**
   3. **Clinical experience**
10. **When choosing insulin regimens for patients with T2DM, what factors influence your insulin initiation practices? Please rank options in order of preference; use 1 for your most preferred option and 4 for your least preferred option.**
    1. **Clinical experience**
    2. **Information from peers**
    3. **Manufacturer information**
    4. **CME education**
